# Altered Expression of 14-3-3ζ Protein in Spinal Cords of Rat Fetuses with Spina Bifida Aperta

**DOI:** 10.1371/journal.pone.0070457

**Published:** 2013-08-06

**Authors:** Li-na Wu, Xiao-wei Wei, Yang Fan, Jia-ning Miao, Li-li Wang, Yi Zhang, Di Wu, Zheng-wei Yuan

**Affiliations:** 1 Department of Laboratory Medicine, Shengjing Hospital, China Medical University, Shenyang, PR China; 2 Key Laboratory of Health Ministry for Congenital Malformation, Shengjing Hospital, China Medical University, Shenyang, PR China; CHA University, Republic of Korea

## Abstract

**Background:**

A large number of studies have confirmed that excessive apoptosis is one of the reasons for deficient neuronal function in neural tube defects (NTDs). A previous study from our laboratory used 2-D gel electrophoresis to demonstrate that 14-3-3ζ expression was low in the spinal cords of rat fetuses with spina bifida aperta at embryonic day (E) 17. As a member of the 14-3-3 protein family, 14-3-3ζ plays a crucial role in the determination of cell fate and anti-apoptotic activity. However, neither the expression of 14-3-3ζ in defective spinal cords, nor the correlation between 14-3-3ζ and excessive apoptosis in NTDs has been fully confirmed.

**Methodology/Principal Findings:**

We used immunoblotting and quantitative real-time PCR (qRT-PCR) to quantify the expression of 14-3-3ζ and double immunofluorescence to visualize 14-3-3ζ and apoptosis. We found that, compared with controls, 14-3-3ζ was down-regulated in spina bifida between E12 and E15. Excessive apoptotic cells and low expression of 14-3-3ζ were observed in the dorsal region of spinal cords with spina bifida during the same time period. To initially explore the molecular mechanisms of apoptosis in NTDs, we investigated the expression of microRNA-7 (miR-7), microRNA-375 (miR-375) and microRNA-451 (miR-451), which are known to down-regulate 14-3-3ζ in several different cell types. We also investigated the expression of p53, a molecule that is downstream of 14-3-3ζ and can be down-regulated by it. We discovered that, in contrast to the reduction of 14-3-3ζ expression, the expression of miR-451, miR-375 and p53 increased in spina bifida rat fetuses.

**Conclusions/Significance:**

These data suggest that the reduced expression of 14-3-3ζ plays a role in the excessive apoptosis that occurs in spina bifida and may be partly regulated by the over-expression of miR-451 and miR-375, and the consequent up-regulation of p53 might further promote apoptosis in spina bifida.

## Introduction

Spina bifida is a complex congenital anomaly of the central nervous system arising from the incomplete fusion or non-fusion of the caudal neuropore of the neural tube. In spite of the introduction of fetal surgical treatments for spina bifida, patients still show neurological deficits of varying degrees, such as sensory and motor weakness in the leg and bowel and bladder incontinence, and require long-term care and assistance [1]. The neurological dysfunction of spina bifida could result either directly from the primary defect in neurulation or secondarily from injuries caused by the intrauterine environment. For example, exposure of the uncovered neural tissue at the site of the spinal defect to amniotic fluid, which is believed to be toxic, could contribute to neurological dysfunction [2], [3]. Our previous studies have identified deficient motor and sensory spinal cord neurons innervating the levator ani muscle in fetal rats with spina bifida aperta [4], [5]. In addition, apoptosis has been implicated as a critical reason for neurological dysfunction in spina bifida. A previous research has shown an increase in cell death in the neuroepithelium of rat embryos with spina bifida at late embryonic stages [6]. Furthermore, our studies also found excessive apoptosis in the neuroepithelium of the mid-dorsal region of the spinal cord in embryos with spina bifida between embryonic day (E) 11 and E13 [7].

To explore the mechanism of neurological deficits in the spinal cords of rats with spina bifida aperta, we performed a proteomics assay using 2-D gel electrophoresis and discovered that four 14-3-3 isoforms, 14-3-3ζ, 14-3-3ε, 14-3-3β and 14-3-3θ were all down-regulated at E17 in rat fetuses with spina bifida [8]. The 14-3-3 proteins are a large family of highly conserved eukaryotic regulatory molecules that play important roles in many biological processes, especially in the regulation of cell death [9]. Among these four 14-3-3 isoforms, the expression of 14-3-3ζ has been correlated with apoptosis in carcinoma and neurodegenerative diseases. The down-regulation of 14-3-3ζ sensitizes head and neck cancer cells to chemotherapeutic agents through the induction of apoptosis [10]. 14-3-3ζ is critical for the suppression of anoikis in lung cancer cells and is a potential molecular target for anticancer therapeutic treatments [11]. Down-regulation of 14-3-3ζ also results in increased apoptosis of cerebral cortex cells in alcohol-treated C57BL/6 mice [12]. In A53T transgenic mice, decreased expression of 14-3-3ζ protein in cytoplasm may account for neuronal cell death [13]. Thus, we speculated that the down-regulation of 14-3-3ζ in spina bifida may be one of the molecular changes that result in excessive apoptosis. However, altered expression of 14-3-3ζ during embryonic development and the link between 14-3-3ζ and excessive apoptosis have not been reported in spina bifida.

Recently, more and more studies have begun to address the regulation of 14-3-3ζ and the mechanism by which 14-3-3ζ inhibits apoptosis. MicroRNAs (miRNAs) that regulate 14-3-3ζ have been reported in the literature. In glioma cells, the over-expression of microRNA-7 (miR-7) down-regulated 14-3-3ζ [14]. In K562 cells, up-regulated expression of microRNA-375 (miR-375) was associated with down-regulated expression of 14-3-3ζ [15]. The repression of 14-3-3ζ by the regulatory microRNA-451 (miR-451) has been confirmed under conditions of defective erythroid differentiation and erythroid oxidative stress [16], [17]. The influence of 14-3-3ζ protein on the apoptotic machinery occurs at multiple levels of regulation. Many studies have shown that 14-3-3ζ protein can bind numerous effectors of apoptosis to inhibit their pro-apoptotic function. Such effectors include, for example, the BH3 domain-containing proteins BAD and BAX, as well as key signaling components and transcription factors involved in the apoptotic response, such as FOXO, A20, and ASK1 [18], [19], [20], [21], [22], [23], [24]. As one of the transcriptional regulators of BH3 domain-containing proteins, p53 plays a crucial role in apoptosis by promoting the synthesis of these proteins [25], [26]. It has been reported that 14-3-3ζ over-expression increased the degradation of p53 in breast cancer. Conversely, the down-regulation of 14-3-3ζ by siRNAs was shown to lead to increased p53 protein expression [27].

Therefore, in this paper, we confirmed the expression of 14-3-3ζ in spinal cords from normal rat fetuses and fetuses with spina bifida from E11 to E19, and selected miR-7, miR-375 and miR-451 as upstream regulators and p53 as the downstream effector of 14-3-3ζ. We discovered that 14-3-3ζ was down-regulated between E12 and E15 in spina bifida. Low expression of 14-3-3ζ and excessive apoptotic cells were simultaneously observed in the dorsal region of spinal cords with spina bifida. Accompanying the reduced expression of 14-3-3ζ, miR-375, miR-451 and p53 were all increased. Thus, we speculated that the low expression of 14-3-3ζ, which may be regulated by the over-expression of miR-451 and miR-375, and the consequent up-regulation of p53 may contribute to excessive apoptosis in spina bifida.

## Results

### All-trans-retinoic Acid (atRA) Induces Neural Tube Defects (NTDs) in Rats

**Figure 1:**
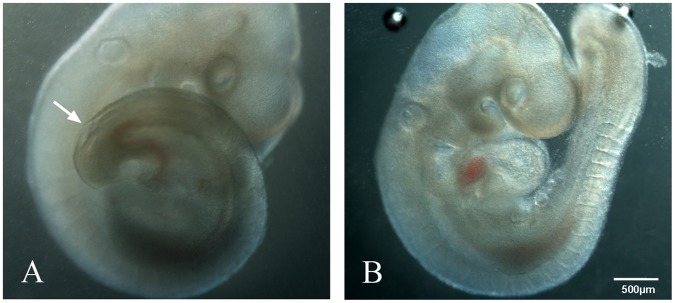
Models of spina bifida or control rat fetuses. Rat embryos (E11) obtained from atRA-treated (A) and control (B) pregnancies. The embryos from atRA-treated pregnancies exhibited spina bifida aperta (arrow).

In the atRA-fed group, 265 live embryos were harvested from 52 pregnant rats from E11 to E19. Gross morphologic examination under a stereomicroscope showed spina bifida aperta in the lumbosacral region (47.2%, 125) (**Figure 1**). In the control group, 165 embryos were harvested from 28 dams. None of the control embryos showed spina bifida.

### Expression of 14-3-3ζ in Posterior Spinal Cords of Normal Rat Fetuses

**Figure 2:**
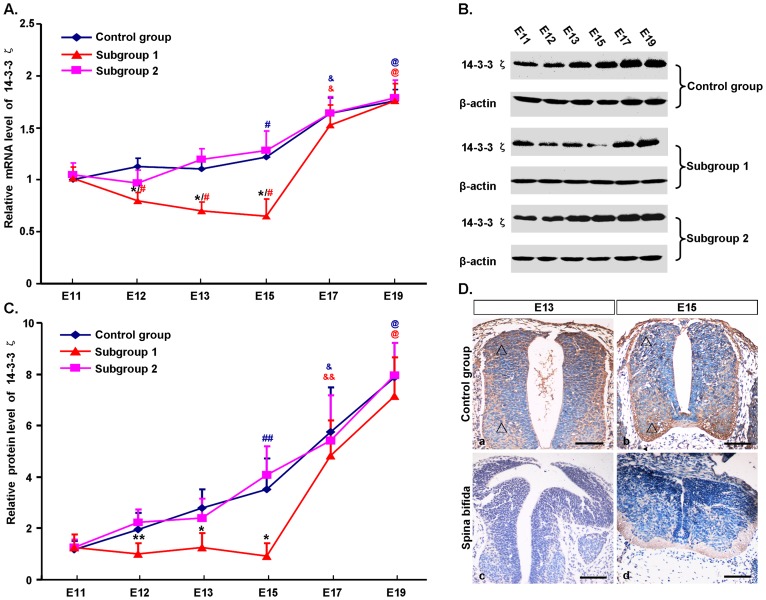
Spatiotemporal expression of 14-3-3ζ in the posterior spinal cords of rat embryos between E11 and E19 obtained using qRT-PCR (A), immunoblot (B and C) and immunohistochemistry (D). **A,** comparison of 14-3-3ζ mRNA expression in the control group (blue), subgroup 1 (red) and subgroup 2 (pink) by qRT-PCR. The control group consisted of rat fetuses without atRA treatment, subgroup 1 those with spina bifida aperta induced by atRA treatment, and subgroup 2 those treated with atRA but without spina bifida aperta. **B and C,** immunoblot results of 14-3-3ζ protein. Analysis of 14-3-3ζ protein was expressed with β-actin as the internal control. Quantification of immunoblot results showed the relative density of 14-3-3ζ protein. **D,** transverse sections of spinal cords stained for 14-3-3ζ protein (brown) in control embryos (a, b) and atRA-induced spina bifida embryos (c, d) at E13 and E15. Sections were counterstained with hematoxylin (blue). In controls, the expression of 14-3-3ζ in the dorsal and ventrolateral part of the spinal cord (triangle) was higher than in the central canal region. At E15, the sections collected were more caudal than at E13, so the spinal cords at E15 appear smaller. The Y-axis represents the relative level of 14-3-3ζ, and the X-axis represents different gestational days. ** *P*<0.05 and * *P*<0.01 vs. control group; ## *P*<0.05 and # *P*<0.01 vs. E11; && *P*<0.05 and & *P*<0.01 vs. E11, E12, E13 and E15; @ *P*<0.01 vs. E11, E12, E13, E15 and E17; blue symbols indicate the control group; red symbols indicate subgroup 1. Scale bars = 100 µm.

Overall, the expression of 14-3-3ζ in normal rat fetuses gradually increased with embryonic development between E11 and E19, peaking at E19. Specifically, 14-3-3ζ mRNA levels (**data shown in** **table 1**) remained constant with no significant changes from E11 to E13. At E15, it significantly increased from the level at E11 (1.175±0.087 vs. 0.966±0.073, *P*<0.01). From E15 to E17, 14-3-3ζ mRNA levels increased notably and continued to increase significantly until E19 (**Figure 2** **, A - blue line**). Immunoblot analysis demonstrated that protein expression levels changed in a slightly different way than 14-3-3ζ mRNA levels. From E11 to E15, 14-3-3ζ protein levels increased slowly, while between E15 and E19 they increased sharply (**Figure 2** **, B - control group, C - blue line, data shown in** **table 2**). At E15, 14-3-3ζ protein levels had increased significantly compared to the levels at E11 (3.508±1.201 vs. 1.175±0.331, *P*<0.05). The 14-3-3ζ protein was mainly distributed in the periphery of the spinal cord, especially ventrally and dorsally (**Figure 2** **, D - a, b**).

**Table 1:** Temporal expression of 14-3-3ζ mRNA in posterior spinal cords of rat fetuses.

	Control group	Subgroup 1	Subgroup 2
E11	0.966±0.073	0.978±0.106	1.012±0.112
E12	1.091±0.077	0.772±0.074* ^/#^	0.936±0.123
E13	1.065±0.083	0.678±0.081* ^/#^	1.155±0.102
E15	1.175±0.087#	0.629±0.156* ^/#^	1.237±0.182#
E17	1.583±0.145 &	1.476±0.187 &	1.585±0.153&
E19	1.700±0.107^ @^	1.699±0.159 ^@^	1.730±0.164^@^

Control group indicates rat fetuses without atRA treatment, and subgroup 1 indicates those with spina bifida aperta by atRA treatment, subgroup 2 those without spina bifida aperta by atRA treatment.

* *P*<0.01 vs. control group;

# *P*<0.01 vs. E11;

& *P*<0.01 vs. E11, E12, E13 and E15;

@P<0.01 vs. E11, E12, E13, E15 and E17.

**Table 2:** Temporal expression of 14-3-3ζ protein in posterior spinal cords of rat fetuses.

	Control group	Subgroup 1	Subgroup 2
E11	1.175±0.331	1.263±0.512	1.268±0.291
E12	1.968±0.625	1.015±0.425**	2.234±0.513
E13	2.805±0.724	1.265±0.564*	2.413±0.732
E15	3.508±1.201##	0.925±0.496*	4.073±1.134^ #^
E17	5.745±1.733^&^	4.823±1.397&&	5.423±1.753$
E19	7.883±1.345^@^	7.165±1.516 ^@^	7.955±1.264^@^

** *P*<0.05 and *P<0.01 vs. normal control;

## *P*<0.05 and ^#^ *P*<0.01 vs. E11;

&& *P*<0.05 and ^&^ *P*<0.01 vs. E11, E12, E13 and E15;

@P<0.01 vs. E11, E12, E13, E15 and E17;

$ *P*<0.05 vs. E11, E12, E13.

### 14-3-3ζ is Reduced in the Spinal Cords of Rat Embryos with Spina Bifida Aperta

The developmental change in 14-3-3ζ expression in spina bifida (**Figure 2** **, A - red line, B - subgroup 1, C - red line, D - c, d**) was notably different from the change that occurred in normal fetuses, especially at early stages. Compared with the constant or steadily increasing expression of 14-3-3ζ between E11 and E15 observed in normal embryos, 14-3-3ζ expression significantly decreased in embryos with spina bifida. Specifically, compared with controls, both 14-3-3ζ mRNA and protein levels were significantly decreased in spina bifida rat fetuses between E12 and E15, with no significant difference at E11, E17 or E19 (**data shown in** **table 1** **and** **2**). Compared with normal fetuses, the reduced expression of 14-3-3ζ protein in spina bifida was concentrated in the ventral and dorsal regions of the spinal cord (**Figure 2** **, D**). To avoid the influence of atRA itself on the change in 14-3-3ζ expression, we included spinal cords from rat fetuses harvested from atRA-treated pregnant rats that did not exhibit spina bifida as an another experimental group (subgroup 2). In this group, the expression of 14-3-3ζ was similar to that of normal rat fetuses (**Figure 2** **, A - pink line, B - subgroup 2, C - pink line, data shown in** **table 1** **and** **2**).

### Over-expression of miR-375 and miR-451 in Defective Spinal Cords

**Figure 3:**
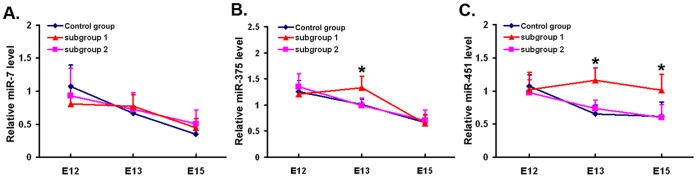
Expression of miR-7, miR-375 and miR-451 in spinal cords of rat fetuses. Expression of miR-7 (A), miR-375 (B) and miR-451 (C) in spinal cords of control group (blue), subgroup 1 (red) and subgroup 2 (pink) embryos at E12, E13 and E15 detected by qRT-PCR. The Y-axis represents relative level of miRNA and the X-axis represents different gestational days. * *P*<0.01 vs. control group.

To initially explore the mechanism by which 14-3-3ζ is down-regulated in spina bifida, we measured miR-7, miR-375 and miR-451 (upstream regulators of 14-3-3ζ) in normal and defective spinal cords at E12, E13 and E15. In subgroup 2, the expression of miR-7, miR-375 and miR-451 was similar to that in controls (**Figure 3** **, A - miR-7, B - miR-375, C - miR-451**). However, in subgroup 1, the expression of miR-375 and miR-451 was significantly different from control fetuses. Compared with controls, the expression of miR-375 was significantly increased at E13 in spina bifida fetuses (1.328±0.218 vs. 1.005±0.098, *P*<0.01) (**Figure 3** **, B**). Up-regulated expression of miR-451 was also observed in spina bifida (E13∶1.163±0.189 vs. 0.652±0.133, E15∶1.012±0.241 vs. 0.617±0.218, *P*<0.01) (**Figure 3** **, C**).

### Reduced 14-3-3ζ is Related to Excessive Apoptosis in Spinal Cords with Spina Bifida

**Figure 4:**
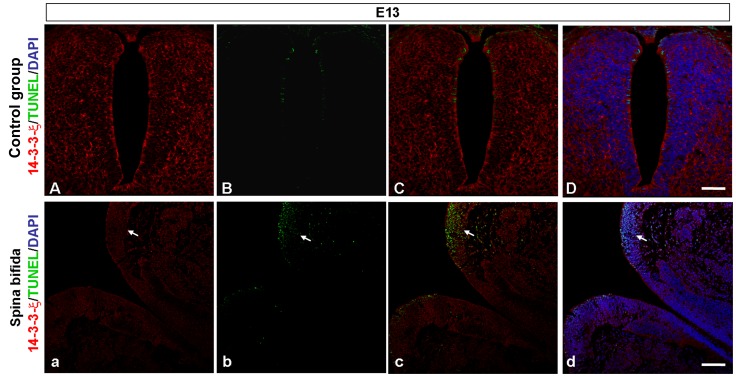
Double immunofluorescent staining for TUNEL and 14-3-3ζ. Double immunofluorescent staining for TUNEL (green) and 14-3-3ζ (red) in lumbo-sacral spinal cords of control embryos (A, B, C, D) and atRA-induced spina bifida embryos (a, b, c, d) at E13. Sections were counterstained with DAPI (blue). The region with increased apoptosis is simultaneous with decreased 14-3-3ζ expression (white arrowheads). Scale bars = 50 µm.

To clarify the relationship between the reduced expression of 14-3-3ζ and excessive apoptosis in spina bifida rat fetuses, we used double immunofluorescence staining for TUNEL and 14-3-3ζ at E13. In both normal and spina bifida rat fetuses, apoptotic cells were concentrated in the dorsal region of the spinal cord. Compared with normal spinal cords, defective spinal cords showed a notable increase in the number of apoptotic cells and a significant reduction in the expression of 14-3-3ζ in the dorsal region (**Figure 4**).

### Up-regulating Expression of p53 in Defective Spinal Cords

**Figure 5:**
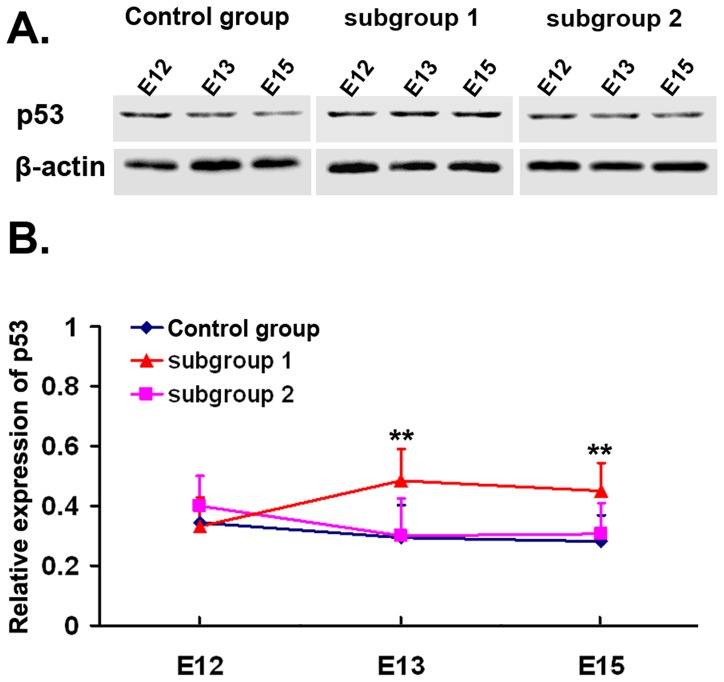
Expression of p53 protein in spinal cords detected by immunoblot. **A and B,** immunoblot results of p53 protein. Analysis of p53 protein was expressed with β-actin as the internal control. Quantification of p53 protein showed the relative density in control group (blue), subgroup 1 (red) and subgroup 2 (pink) embryos at E12, E13 and E15. ** *P*<0.05 vs. control group.

In control and subgroup 2 fetuses, p53 expression was constant, and there was no significant difference between them at E12, E13 and E15 (**Figure 5** **, A - control group, subgroup 2, B - blue line for control group, pink line for subgroup 2**). In contrast, in defective spinal cords, the expression of p53 increased with embryonic development (**Figure 5** **, A - subgroup 1, B - red line**). Compared with control fetuses, spina bifida fetuses showed a significant increase at E13 and E15 (E13∶0.485±0.106 vs. 0.295±0.109, E15∶0.450±0.093 vs. 0.280±0.088, *P*<0.05) and no significant difference at E12.

## Discussion

NTDs are common human birth defects that occur in approximately 1 birth per 1000 worldwide and 2.74 births per 1000 in China [28], [29]. The most common type of NTDs is spina bifida. Despite the clinical application of fetal surgical treatments for spina bifida, therapeutic effects for spina bifida are still unsatisfactory, with neurological complications being the main problem. Thus, researching the mechanisms of neurological dysfunction is essential for developing effective treatments for spina bifida. Maternal administration of atRA has long been used to induce an experimental model of NTDs in the fetal rat [30]. In this study, we used atRA administration to induce spina bifida in our rat model and found 47.2% of fetuses with spina bifida aperta (subgroup 1) and 52.8% without (subgroup 2). This finding is consistent with our previous results [7].

The members of the 14-3-3 protein family are conserved acidic proteins, with seven different isoforms found in mammals (ζ, ε, β, θ, γ, η, and σ) [31]. They function as molecular adapters that interact with key signaling molecules and thereby regulate various cell functions [32], [33], [34]. The 14-3-3 proteins are ubiquitously expressed, but are most abundant in the central nervous system [35]. As a member of the 14-3-3 protein family, 14-3-3ζ has been implicated in tumorigenesis and neural damage of the brain, and in these diseases 14-3-3ζ exhibits anti-apoptotic activity [36], [37]. However, neither its altered expression in the spinal cords during embryonic development, nor the molecular interactions between 14-3-3ζ and excessive apoptosis in spina bifida have been addressed. Thus, in this study, we investigated the expression of 14-3-3ζ in the spinal cords of normal and spina bifida rat fetuses from E11 to E19 using immunoblotting and quantitative real-time PCR (qRT-PCR). We also evaluated the link between 14-3-3ζ and apoptosis using double immunofluorescence and preliminarily explored the molecular mechanisms of apoptosis using qRT-PCR for miR-7, miR-375 and miR-451 (upstream regulators of 14-3-3ζ) and immunoblotting for p53 (a downstream effector of 14-3-3ζ).

Our results demonstrated that in control rat fetuses, 14-3-3ζ protein was abundant in the spinal cord between E11 and E19, increased with embryonic development, and was mainly located in the periphery of the spinal cord. However, the developmental change in 14-3-3ζ expression in spina bifida was notably different from that in control fetuses. Compared with the steadily increasing expression of 14-3-3ζ protein between E11 and E15 observed in control embryos, 14-3-3ζ expression decreased in embryos with spina bifida. The reduced expression of 14-3-3ζ protein in spina bifida was concentrated in the dorsal and ventral regions of the spinal cord, where neuroepithelial cells and sensory and motor neurons of the spinal cord, respectively, are concentrated. It was reported that atRA can down-regulate 14-3-3ζ expression in acute promyelocytic leukemia cells [38]. In this study, to explore the link between reduced 14-3-3ζ expression and atRA in spina bifida aperta, we included another experimental group (subgroup 2), which consisted of spinal cords from morphologically normal fetuses from atRA-treated pregnant rats. In this group, the expression of 14-3-3ζ was similar to that of control rat fetuses, indicating that the reduction of 14-3-3ζ in rat fetuses with spina bifida aperta resulted from the spinal cord defects or injuries by the intrauterine environment, not from the atRA itself. Further research is required to determine whether the regulation of 14-3-3ζ expression by atRA is variable across different cell types.

It has been reported that the key period of neural development in the spinal cords of rats is from E12 to E16. Most of the dorsal root ganglion (DRG) neurons of the muscle and intercostal nerves are generated early, with developmental peaks at E13, while those of the cutaneous and visceral afferent nerves develop later, peaking at E14 [39]. It was reported that the first fibers appear at the lumbar ventral horn and DRGs at E12, with fibers originating from regions L3 to L5 reaching the epidermis of the most distal toes by E16 [40]. In the spinal cord, both sensory and motor neurons are derived from neuroepithelial cells. Therefore, precise regulation of apoptosis in neuroepithelial cells plays a significant role in spinal cord development. It is well known that excessive apoptosis plays a crucial role in neurological deficiency in spina bifida. Compared with normal embryos, the proportion of apoptotic cells in the neuroepithelium increased at E12, E13, and E15 in spina bifida embryos. Apoptotic cells in both normal and spina bifida fetuses were primarily located in the dorsal region of the spinal cord [6], [7]. Coincident with the excessive apoptosis, the expression of 14-3-3ζ protein in spina bifida was significantly lower than that in normal rat fetuses at E12, E13, and E15. And we also confirmed that excessive apoptotic cells and down-regulated 14-3-3ζ expression were simultaneously observed in the dorsal region of spinal cords. The relationship between 14-3-3ζ and apoptosis has also been confirmed in carcinoma, neurodegenerative diseases, and other contexts. For example, over-expression of 14-3-3ζ has been found to promote cell proliferation and inhibit cell apoptosis in breast cancer, lung cancer, and head and neck squamous cell carcinomas, and it may be a potential molecular target for anticancer therapeutic development [10], [11], [27], [41], [42], [43]. In Parkinson’s disease (PD), reduced levels of 14-3-3ζ in neurons may account for loss of dopaminergic neurons [44]. The down-regulation of 14-3-3ζ protein levels results in increased apoptosis of cerebral cortex cells in alcohol-treated C57BL/6 mice [12]. Thus, we postulated that reduced 14-3-3ζ was one of the molecules leading to excessive apoptosis in rat fetuses with spina bifida aperta.

Regulating the balance between survival and apoptotic signaling is a key aspect of cell fate decisions, and 14-3-3 protein contributes to this process. An emerging role for 14-3-3ζ as an effector of pro-survival signaling is suggested in part by the large number of 14-3-3ζ binding proteins involved in apoptosis. 14-3-3 binding inactivates numerous pro-apoptotic proteins, such as the BH3 domain-containing proteins (BAD, BAX, etc.) by sequestering them from their sites of action [45], [46], [47], [48], [49], [50]. p53 is an important transcriptional regulator of BH3 domain-containing proteins and plays a crucial role in apoptosis by promoting the synthesis of these proteins [25], [26]. Thus, to maintain the balance between apoptotic and survival signals, the level and activity of p53 protein must be tightly regulated in cells [51]. In unstressed cells, the half-life of p53 protein is short because the protein is targeted for degradation in the cytoplasm. This process is mainly regulated by the E3 ligase MDM2 [52]. MDM2 binds and ubiquitinates p53, whereby p53 is exported to the cytoplasm and degraded by the proteosome [53]. It has been reported that in breast cancer, 14-3-3ζ over-expression increases the MDM2-dependent proteasomal degradation of p53 via the activation of PI3K-Akt. Conversely, down-regulating 14-3-3ζ expression in 10A. 14-3-3ζ cells via siRNA, leads to increased protein expression of p53 [27]. However, it is unknown whether the regulation of p53 by 14-3-3ζ still takes place in spinal bifida aperta. Here, we measured p53 protein at E12, E13, and E15 in rat fetuses with or without spina bifida aperta, and found that p53 was significantly increased at E13 and E15 in spina bifida fetuses as compared with control fetuses, but p53 was almost the same in subgroup 2 fetuses as in controls from E12 to E15. These data indicate that over-expression of p53 in spina bifida fetuses may result from the reduced 14-3-3ζ, and up-regulation of p53 would facilitate apoptosis. As for the regulatory mechanism of 14-3-3ζ on p53 and the other apoptotic mechanisms influenced by 14-3-3ζ, more experiments are needed.

MicroRNAs (miRNAs) are a class of small (approximately 22 nt), single-stranded, non-coding RNAs that regulate the stability or translational efficiency of their target mRNAs through antisense complementarity to 3′-untranslated regions (UTR) [54]. MiRNAs play critical regulatory roles in various physiological and pathological processes, including differentiation, proliferation, development, apoptosis and oncogenesis [55]. Currently, approximately 800 miRNAs have been identified in the human genome, and approximately 74–92% of the genes are regulated by miRNAs. Certain miRNAs have tissue- or disease-specific expression profiles [56], [57]. The regulation of 14-3-3ζ by miRNAs has been reported in the literature. It was reported that ectopic over-expression of miR-7 in glioma cells can down-regulate 14-3-3ζ, indicating that 14-3-3ζ may be a downstream effector of miR-7 [58]. In Doxorubicin (DOX)-induced senescent K562 cells, the up-regulation of miR-375 was associated with down-regulated expression of 14-3-3ζ, indicating that 14-3-3ζ was a downstream effector of miR-375 [15]. Transfection of gastric cancer cells with miR-375 induces the significant down-regulation of 14-3-3ζ expression and a marked reduction in cell viability. Further analysis showed that miR-375 targets the 3′-UTR of 14-3-3ζ [59]. Except for defective erythroid differentiation and erythroid oxidative stress, the repression of 14-3-3ζ by miR-451 has also been confirmed in early diabetic nephropathy and breast cancer [60], [61]. Therefore, we selected miR-7, miR-375 and miR-451 as the upstream regulators of 14-3-3ζ and measured them at E12, E13 and E15 to confirm the correlation between miRNAs and 14-3-3ζ. We found that, as compared with control fetuses, the expression of miR-375 in spina bifida was significantly up-regulated at E13, and miR-451 was increased at E13 and E15. However, the expression of miR-7, miR-375 and miR-451 in subgroup 2 was similar to that in control fetuses. These data suggest that the low expression levels of 14-3-3ζ in spina bifida aperta may be partially regulated by miR-375 and miR-451. Further investigation is necessary to determine why 14-3-3ζ is expressed at low levels at E12 in spina bifida and how 14-3-3ζ is regulated by miR-375 and miR-451 at E13 and E15.

In conclusion, in this study, we found a significant reduction in the expression of 14-3-3ζ in spina bifida rat fetuses between E12 and E15. Low 14-3-3ζ expression levels were observed during the same time period, with excessive apoptotic cells in the dorsal region of spinal cords with spina bifida. Furthermore, we showed that, in contrast to the reduction of 14-3-3ζ expression, the levels of miR-451, miR-375 and p53 increased in spina bifida rat fetuses. Therefore, we speculated that the low expression of 14-3-3ζ might be regulated in part due to the over-expression of miR-451 and miR-375, and the over-expression of p53 may be caused by the down-regulation of 14-3-3ζ in spina bifida aperta. This may be a pathway underlying excessive apoptosis in spina bifida.

## Materials and Methods

### 

#### IRB: NO.2013PS112K

All animals in this study were from animal center of Shengjing hospital in China Medical University. Pregnant female rats were anesthetized and killed by an overdose injection of 10% chloral hydrate into the abdominal cavity. All studies were performed in accordance with the protocol approved by the Institutional Animal Care and Use Committee of the China Medical University for Basic Research in Developmental Disabilities. All surgeries were performed under anesthesia, and all efforts were made to minimize suffering.

### Animals and Preparation of Spinal Cords

Outbred 10–12 week old Wistar rats (250–300 g) were purchased from the animal center of China Medical University. The animals were maintained in a temperature- (20–24°C) and humidity- (50–70%) controlled environment with a 12 h light/dark cycle. Solid laboratory chow and water were available ad libitum. The appearance of vaginal plugs in the female rat the morning after mating was considered E0. We randomly divided 80 pregnant rats into 2 groups for treatment (n = 52 atRA treat group, n = 28 control group). In the experimental group, spina bifida aperta was induced with a single intragastric administration of all-trans retinoic acid (atRA) (Sigma; 4% [wt/vol] in olive oil; 140 mg/kg body weight) via gavage feeding at E10 as previously described [62]. Control group was treated with the same amount of olive oil on the same day.

Pregnant rats were killed at E11, E12, E13, E15, E17 or E19 by an overdose injection of 10% chloral hydrate into the abdominal cavity. Fetuses were then harvested. After examining the malformations under a microscope, embryos from the atRA treatment group were divided into 2 subgroups: fetuses with and without spina bifida aperta, named subgroup 1 and subgroup 2 respectively. At E11, E12 and E13, the posterior spinal cords were isolated from the 11th pair of somites to the tail buds. At E15, E17 and E19, the spinal cords were dissected from the inferior margin of the forelimb buds to the tail buds. For immunoblotting and real-time quantitative PCR analysis, 16 posterior spinal cords were isolated from each of the 2 subgroups and from the control group at each timepoint (8 from each group were used for immunoblotting and 8 for qRT-PCR) and stored at −80 °C. At each timepoint, embryos in each group were harvested from at least 4 independent dams. For immunohistochemistry and immunofluorescence, 3 spina bifida embryos and 3 control embryos at E13 and E15 were fixed in freshly prepared 4% paraformaldehyde (in PBS) and subsequently processed and embedded in paraffin. Each of the embryos used for immunohistochemistry and immunofluorescence was obtained from a different dam. Serial transverse sections (2.5 µm) were cut through the lumbo-sacral regions with a microtome (Thermo, Walldorf, Germany).

### RNA Isolation

The total RNA of the posterior spinal cord was isolated using a mirVana™ miRNA Isolation Kit (Ambion, USA) according to the manufacturer’s protocol. RNA concentration was determined using a spectrophotometer (NanoDrop ND1000, NanoDrop Technologies Inc, USA). RNA with an A260 nm/A280 nm ratio less than 1.8 was discarded.

### Quantification of mRNA by qRT-PCR

For analyzing the expression of 14-3-3ζ mRNA (accession number: NM_013011.3; Primer sequences: forward 5′-GAGTCGTACAAAGACAGCACGC TAA-3′, reverse 5′-GTGGGACAGCATGGATGACAA-3′), total RNA (3 µg) was reverse-transcribed into cDNA at 37°C for 15 min, 85°C for 5 sec., and then at 4°C with random 6-mer oligos (50 pmol), oligo dT primer (25 pmol) and the PrimeScript RT reagent kit (Takara) in 20 µl of reaction solution. Diluted cDNA (1∶10) was subjected to qRT-PCR using a SYBR® Premix Ex Taq™ II kit (Takara, Japan) in 20 µl of reaction solution that contained 2 µl of cDNA templates, 0.4 µM of each primer and 10 µl of 2**×**SYBR Green Master Mix, brought to final volume with RNase-free water. The reactions were performed in triplicate on a PCR thermal cycler (7500 Fast Real-time PCR System, Applied Biosystems, USA). PCR was performed as follows: pre-denaturation at 95°C for 30 sec, 45 cycles of denaturation at 95°C for 5 sec, and annealing at 60°C for 20 sec. The negative control (without reverse transcriptase) gave no signal. The relative mRNA levels of each sample were calculated by the 2^−ΔΔct^ method and expressed as a fold induction relative to the control spinal cords at E11 following ß-actin (accession number: NM_031144; Primer sequences: forward 5′- GGAGATTACTCCCTGGCTCCTA-3′, reverse 5′- GACTCATCGTACTCCTGCTT GCTG-3′) normalization.

### Quantification of miRNA by qRT-PCR

To evaluate the expression of miR-7, miR-375 and miR-451 in the posterior spinal cord at E12, E13 and E15, qRT-PCR was carried out using the following TaqMan MicroRNA assays (Applied Biosystems, USA): rno-miR-7 (assay ID 000582), hsa-miR-375 (assay ID 000564), mmu-miR-451 (assay ID 001141) and U6 snRNA (assay ID 001973; used as the endogenous control gene). U6 (as the endogenous control gene), miR-7, miR-375 and miR-451 cDNA was reverse-transcribed from the total RNA (obtained from the same sample used for the mRNA expression assays) using specific primers and a TaqMan® MicroRNA Reverse Transcription Kit (Applied Biosystems, USA) in accordance with the manufacturer’s instructions. The reaction mixture consisted of 5 µL total RNA, 1.5 µL 10× RT buffer, 3 µL 5× RT primer, 0.15 µL dNTPs (100 mM), 0.19 µL RNase Inhibitor (20 U/µL), 4.16 µL Nuclease-free water, and 1 µL MultiScribe™ Reverse Transcriptase (50 U/µL) in a final reaction volume of 15 µL. Reaction mixtures were incubated at 16°C for 30 min, 42°C for 30 min, 85°C for 5 min and then held at 4°C. An Applied Biosystems 7500 Fast Real-Time PCR System was used to carry out qRT-PCR. The reaction mixture for real-time PCR consisted of 1.3 µL template cDNA, 10 µL TaqMan 2× Universal PCR Master Mix (Applied Biosystems, USA), 7.7 µL Nuclease-free water, and 1 µL 20× TaqMan MicroRNA Assay in a total reaction volume of 20 µL. Reactions were performed in triplicate on a PCR thermal cycler (7500 Fast Real-time PCR System, Applied Biosystems, USA). PCR was performed as follows: UTPase inactivation at 50°C for 2 min, pre-denaturation at 95°C for 10 min, 45 cycles of denaturation at 95°C for 15 sec and annealing at 60°C for 60 sec. The negative control (without reverse transcriptase) gave no signal. The relative quantities of miRNA were calculated using the 2^−ΔΔct^ method and expressed as a fold induction compared with control spinal cords at E12 following U6 normalization.

### Immunoblot Analysis

Proteins from the posterior spinal cords were isolated in lysis buffer (Beyotime, P0013B, China) and quantified using a protein assay kit (Beyotime, P0010, China). Aliquots of protein extracts (15 µg per sample for 14-3-3ζ and 70 µg per sample for p53) were heated with sample buffer and separated using 12.5% SDS-PAGE and then transferred with Tris–HCl methanol (20 mM Tris, 150 mM glycine and 20% methanol) onto polyvinylidene difluoride membranes (Millipore, USA) in a trans-blot electrophoresis transfer cell (Bio-Rad). Blots were probed with the primary antibody: rabbit anti-14-3-3ζ (Santa Cruz Biotechnology sc-1019), sheep anti-p53 (Abcam incorporation ab16121) or goat anti-actin (Santa Cruz Biotechnology sc-1616) overnight at 4°C. After being washed with PBS containing 0.05% Tween 20, membranes were incubated for 2.5 h at room temperature with anti-rabbit, anti-sheep or anti-goat IgG antibody (CWBIO) conjugated with horseradish peroxidase. Immunopositive bands were visualized using enhanced chemiluminescence reagents (ECL, GE healthcare). Detected bands were quantified with Gel-pro4.0 software (Media Cybernetics, LP). The relative density of each protein was calculated by dividing the optical density value of each protein by that of the loading control (β-actin).

### Immunohistochemical Analysis

Immunohistochemical staining for 14-3-3ζ was performed on transverse sections of the lumbo-sacral spinal cord in E13 and E15 embryos. Sections were de-waxed in xylol, rehydrated in decreasing concentrations of alcohol, and then subjected to microwave antigen retrieval (10 min in 0.1 M citrate acid buffer solution, pH 6). Sections were blocked with 0.3% hydrogen peroxide and PBS containing 10% fetal calf serum (FBS) and 0.1% Triton x-100. Sections were then incubated with a rabbit antibody against 14-3-3ζ (1∶100) (Santa Cruz Biotechnology sc-1019) in 10% FBS overnight at 4°C. After washing, sections were incubated with peroxidase-conjugated goat anti-rabbit IgG (Boster) in 10% FBS containing 0.1% Triton x-100 for 20 min at room temperature and colored using Diaminobenzidine (DAB). Images were taken with a microscope (Nikon, Japan).

### Double Immunofluorescent Staining for TUNEL and 14-3-3ζ

Serial transverse sections of the embryonic lumbo-sacral spinal cord underwent TUNEL analysis and immunofluorescence for 14-3-3ζ as previously described [7]. Briefly, after being de-waxed and rehydrated, sections were permeated with 15 µg/ml proteinase K in PBS containing 0.1% Triton x-100 for 10 min at 37°C and then ice cold 10 mM citrate acid containing 0.1% Triton x-100 for 2 min. Then, the sections were blocked with PBS containing 10% FBS and 0.1% Triton x-100 and incubated with 14-3-3ζ antibody (1∶100) at 4°C overnight. After washing, sections were incubated with the TUNEL reaction mixture and TRITC-anti-rabbit IgG at 37°C for 1.5 h in the dark and counterstained with DAPI for 5 min before being mounted on slides. Images were taken with a C1 confocal microscope (Nikon, Japan).

### Statistical Analysis

Data, including the 2^−ΔΔct^ value of each sample for qRT-PCR and the relative density of bands for immunoblot analysis, are expressed as the mean ± SD. Data are parametric. One-way ANOVA was used to evaluate differences in 14-3-3ζ expression (both in mRNA and protein levels), miR-7, miR-375, miR-451 and p53 protein expression among the controls, subgroup 1 and subgroup 2. If a significant *P*-value was reached, the SNK post-hoc test was used for pairwise comparisons to identify which means were significantly different from one another. Similarly, the different expression levels of 14-3-3ζ, miR-7, miR-375, miR-451 and p53 among multiple developmental stages were also evaluated by one-way ANOVA and the SNK post-hoc test. *P*<0.05 was considered statistically significant.
